# Draft Genome Sequence of a Benzo[*a*]pyrene-Degrading Bacterium, *Olleya* sp. Strain ITB9

**DOI:** 10.1128/genomeA.01328-15

**Published:** 2015-11-12

**Authors:** Masahiko Okai, Akihiro Watanabe, Masami Ishida, Naoto Urano

**Affiliations:** Department of Ocean Sciences, Graduate School of Marine Science and Technology, Tokyo University of Marine Science and Technology, Tokyo, Japan

## Abstract

*Olleya* sp. ITB9 is a benzo[*a*]pyrene-degrading bacterium, isolated from surface water near a waste treatment plant at Tokyo Bay, Japan. Here, we present the draft genome sequence of this strain, which consists of 58 contigs corresponding to 3.4 Mb and a G+C content of 31.2%.

## GENOME ANNOUNCEMENT

Polycyclic aromatic hydrocarbons (PAHs) are carcinogenic and toxic compounds that have two or more fused aromatic rings. They are widely present in the ground, water, and atmosphere, and they are also produced by the incomplete combustion of hydrocarbons such as gasoline, oil, and coal. PAHs have serious effects on human health and the environment. The five-ring PAH compound benzo[*a*]pyrene (BaP) is one of the most carcinogenic and toxic PAHs. The degradation of BaP by soil bacteria such as *Mycobacterium*, *Sphingomonas*, and *Bacillus* has been studied extensively (1–3). *Olleya* sp. ITB9, a member of the family *Flavobacteriaceae* of the phylum *Bacteroidetes*, was isolated from surface water near a waste treatment plant at Tokyo Bay in Japan and showed the degradation of BaP at a high concentration (4). *Olleya* sp. ITB9 may be a new candidate for the bioremediation of BaP.

We inoculated *Olleya* sp. ITB9 into liquid NB medium (1% Bacto Peptone, 0.5% NaCl, and 0.3% beef extract) containing 3.5% artificial seawater, and cultured it at 120 rpm for 24 h at 25°C. The culture was collected by centrifugation at 3,000 rpm for 10 min at 4°C. Genomic DNA was extracted using a DNA extraction kit, ISOPLANT II (Nippon Gene, Tokyo). We prepared shotgun and 8-kb pair-end libraries and sequenced the genome of *Olleya* sp. ITB9 using the Roche 454-GS junior platform. The reads were *de novo* assembled into contigs using the GS *de novo* assembler (Newbler version 2.9 software). The draft genome comprised 58 contigs, ranging from 484 bp to 223,494 bp with an *N*_50_ value of 103,396 bp and an *N*_90_ value of 30,360 bp. The draft genome was annotated by the Microbial Genome Annotation Pipeline (MiGAP) online server (http://www.migap.org), which uses MetaGeneAnnotator version 1.0 (5), tRNAscan-SE version 1.23 (6), RNAmmer version 1.2 (7), and NCBI BLAST (8). The draft genome annotations predict two rRNA sequences, 36 tRNA sequences, and 3,058 coding sequences (CDSs). The 16S rRNA gene sequence of *Olleya* sp. ITB9 is 99.3%, 96.2%, and 99.5% identical to those of *Olleya* sp. VCSM12, *Olleya* sp. VCSA23, and *Olleya marilimosa* CAM030, respectively. The genome sequences of these three strains have already been deposited at DDBJ/EMBL/GenBank. Our comparison of the genome sequences of *Olleya* sp. ITB9 and the other three strains will provide useful information for the identification of enzymes associated with BaP degradation.

### Nucleotide sequence accession numbers.

The draft genome sequence of *Olleya* sp. ITB9 has been deposited at DDBJ/EMBL/GenBank under the accession number BCBW00000000. The version described in this paper is the first version, BCBW01000000.
